# Prophylactic treatment with flumethrin, a pyrethroid (Bayticol®, Bayer), against *Anaplasma phagocytophilum* infection in lambs

**DOI:** 10.1186/1751-0147-54-31

**Published:** 2012-05-23

**Authors:** Snorre Stuen, Jörg MD Enemark, Karin Artursson, Bent Nielsen

**Affiliations:** 1 Department of Production Animal Clinical Sciences, Norwegian School of Veterinary Science, Kyrkjevegen 332/334, N-4325 Sandnes, Norway; 2 Bayer A/S, DK-2300 København, Denmark; 3 National Veterinary Institute, Uppsala, Sweden

**Keywords:** *Anaplasma phagocytophilum*, Treatment, Pyrethroids, Lamb

## Abstract

**Backgroud:**

*Anaplasma phagocytophilum* (formerly *Ehrlichia phagocytophila*) causes the disease tick-borne fever (TBF) in domestic ruminants and has for decades been one of the main scourges for the sheep industry in the coastal areas of Norway. Current control strategies are based on reduction of tick infestation by chemical acaricides.

**Methods:**

In the present study, we investigated if frequent pour-on applications of pyrethroids would reduce tick infestion rate and seroprevalence of *A. phagocytophilum* infection in sheep. Forty lambs, one month old, of the Norwegian White Sheep breed were used. The lambs belonged to the experimental sheep flock at the Department of Production Animal Clinical Sciences. None of the lambs had been on *I. ricinus* infested pasture before turnout (day 0). All lambs were twins and twenty lambs were treated with a pour-on pyrethroid (Bayticol®, Bayer A/S, DK-2300) with a dose of 5 ml on days 0, 14, 28, 42, 56, 70, 84, 98, 112 and 128. Twenty lambs were untreated controls. The lambs were collected every fourteen days on pasture for treatment. In addition, the lambs were examined for ticks, blood sampled, weighed, and rectal temperature was recorded.

**Results and conclusion:**

A significant reduction in tick infestion rate was detected on treated lambs. However, the present results indicate that frequent acaricide treatment does not reduce the seroprevalence to *A. phagocytophilum* on tick-infested pasture.

## Background

The rickettsia *Anaplasma phagocytophilum* (formerly *Ehrlichia phagocytophila*) causes tick-borne fever (TBF) in domestic ruminants, a disease which has also been diagnosed in several other animal species and human beings [1-3]. In Europe, *A. phagocytophilum* is mainly transmitted by *Ixodes ricinus* ticks. TBF has for decades been one of the main scourges for the sheep industry in the coastal areas of Norway [4]. A serological survey in sheep and wild cervids indicated that *A. phagocytophilum* infection was widespread along the coast of southern Norway [5,6].

Sheep flocks on tick-infested pastures may suffer heavy losses due to direct mortality. The losses may vary from year to year and from area to area [7]. In one study, 79% of the lambs that died one year on tick-infested pastures were infected with *A. phagocytophilum*[8]. However, the severity of disease on *I. ricinus* infested pastures is influenced by several factors, such as questing activity of the ticks, variants of *A. phagocytophilum* in the tick population, prevalence of other tick-transmitted pathogens, and host factors such as age, immune status and body condition of the animal [4].

Current control strategies are based on the reduction of tick infestation by chemical acaricides. In Europe, this is mostly done by pour-on applications of pyrethroids. Normally this treatment has to be repeated several times during the grazing season. The most used pyrethroids in Norway are deltamethrin (Coopersect®, Intervet), flumethrin (Bayticol®, Bayer), cypermethrin (Crovect®, Young) and alphacypermethrin (Dysect®, Fort Dodge) (Legemiddelverket, personal information). Long-acting tetracycline is also used in the UK as a prophylactic measure given before animals are moved from tick-free environment into tick-infested pasture [9,10]. However, there is a growing concern about the environmental safety and human health, increasing cost of chemical control and the increasing resistance of ticks to pesticides [11].

Treatment frequency of pyrethroids has been questioned by farmers and veterinary practitioners for several years. The main reason for the present study was to investigate if frequent treatment with pyrethroids will reduce the tick infestion rate, improve weight gain and lower the prevalence of *A. phagocytophilum* infection in lambs on *I. ricinus* infested pasture.

## Material and methods

### Animals, management and treatment

Forty lambs of the Norwegian White Sheep breed were used. The lambs belonged to the experimental sheep flock at the Department of Production Animal Clinical Sciences. The study was approved by the National Animal Research Authority (Norway). None of the lambs had been on *I. ricinus* infested pasture before turnout (day 0). The lambs were grouped in two according to equal distribution of sex and mean live weight. All lambs were twins and twenty lambs (10 twin couples) were treated with pour-on pyrethroids (Bayticol®, Bayer A/S, DK-2300) with a dose of 5 ml on days 0, 14, 28, 42, 56, 70, 84, 98, 112 and 126. The lambs were treated along the back from neck to tail of each lamb according to the manufacturers recommendation. The lambs were housed indoors for at least two hours post each treatment. In addition, twenty lambs were untreated controls.

The lambs were four-weeks old at turnout (first week of May). After turnout, the lambs were collected every fourteen days for inspection and treatment. On each sampling, all lambs were blood sampled (serum), weighed, and rectal temperature was obtained. In addition, each lamb was examined for ticks, especially on the head, axillae and inguinal regions [12,13].

Gastrointestinal parasites are common on the actual pasture. In order to reduce the influence of *Eimeria* spp. and gastrointestinal nematodes, the lambs were treated with toltrazuril (Baycox®, Bayer) on day 7 and with fenbendazol (Valbazen®, Pfizer) on days 28, 56, 84 and 112. In order to monitor the parasite burden, fecal egg count reduction test (FECRT) was applied on ten randomly selected lambs every second week [14].

### Blood samples and haematology

Full blood samples (EDTA) were collected if fever (≥ 40.5°C) was recorded. Haematological values including total and differential leucocyte counts were determined electronically (Technicon H1®, Miles Inc., USA) and blood smears were prepared and stained with May-Grünwald Giemsa. Four hundred neutrophils were examined on each smear by microscopy, and the number of cells containing *Anaplasma* inclusions was recorded.

### Serology

Sera were collected within four days after birth, at turnout (day 0) and then every second week on pasture, i.e. on days 0, 14, 28, 42, 56, 70, 84, 98, 112 and 126. Sera were analysed using an indirect immunofluorescence antibody assay (IFA) to determine the antibody titre to an equine variant of *A. phagocytophilum* (formerly *Ehrlichia equi*) [5]. Briefly, two-fold dilutions of sera were added to slides precoated with *A. phagocytophilum* antigen (Protatec, St. Paul, Minn.). Bound antibodies were visualized by fluorescein-isothiocyanate (FITC)-conjugated rabbit-anti-sheep immunoglobulin (Cappel, Organon Teknika, West Chester, PA). Sera were screened for antibodies at dilution 1:40. If positive, the serum was further diluted and retested. A titre of 1.6 (log_10_ reciprocal of 1:40) or more was regarded positive.

### Statistics

Statistical calculations were done using Statistix, version 4.0 (Analytical Software). Statistical analyses on ticks, seroconversion and treatment were performed using a chi-square test. A two sample *t* test was used to analyse the lamb weight. A *P* value of <0.05 was considered significant.

## Results

A low gastro-intestinal parasite burden was observed in the lambs during the grazing season (data not shown). Clinical disease or direct losses were not observed in any lamb during the study period, except for fever (>40.5°C) in 26 lambs, whereas high fever (≥41°C) was observed in three lambs. Of these 26 lambs only two and four lambs, including lambs with high fever, were found positive for an *A. phagocytophilum* infection by blood smear investigation in the treated and control group, respectively.

Only 37 ticks (*I. ricinus*) were observed on the lambs, 35 nymphs and 2 adults (Table 1). Most of the ticks were detected in May/June (19) and in August/September (16). Altogether, 7 (19%) and 30 (81%) ticks were found on treated and control lambs, respectively, a significant difference (P < 0.0001) in tick burden (Table 2). These ticks were found on altogether 24 lambs, whereas each lamb was infested with one single tick, except for at two occasions (in controls). Six of these 24 tick-infested lambs (25%) did not seroreact to *A. phagocytophilum.*

**Table 1:** Number of ticks detected on pyrethroid-treated and control lambs after turn-out (day 0)

**Day**	**0**	**14**	**28**	**42**	**56**	**70**	**84**	**98**	**112**	**126**	**Total**
Treated	0	1	1	1	0	1	2	0	0	1	7
Control	0	8	5	3	0	1	4	1	5	3	30
Total	0	9	6	4	0	2	6	1	5	4	37

Each group includes 20 lambs.

**Table 2:** Comparison of the total number of ticks on pyrethroid-treated and control animals on pasture

	**Treatment against ticks**			
**Ticks**		**Yes**	**No**	**Total**
	Yes	7	30	37
	No	173	150	323
	Total	180	180	360

Forty lambs were investigated every fourteen days at nine occasions (360 observations).

Yates corrected χ2 = 14.58 (p = 0.0001).

The serological results are shown in Table 3. A total of 35 lambs (88%) had maternal antibodies at sampling four days after birth. The maternal antibodies gradually decreased and four weeks after turnout only four lambs had antibodies to *A. phagocytophilum*. It is unknown if these were of maternal origin or acquired on pasture. A total of 5 and 10 lambs in the treated and untreated group, respectively, seroconverted (> two-fold increase in antibody titre) (P > 0.05). Ticks were not detected on three (20%) of these lambs. At the end of the study period 14 lambs (35%) were found seropositive, i.e. 6 (30%) in the treated and 8 (40%) in the control group, respectively.

**Table 3:** **Number of seropositive (%) lambs and the antibody titre (mean ± SD) to *A. phagocytophilum *in treated (N = 20) and untreated (N = 20) lamb groups**

	**Day**	−28*	0*	**28**	**56**	**84**	**112**	**126**
Treated	n (%)	18 (90)	13 (65)	3 (15)	0 (0)	0 (0)	1 (5)	6 (30)
	Titre	2.3 ± 0.49	2.0 ± 0.35	1.6 ± 0.0	<1.6	<1.6	3.1 ± 0.0	2.7 ± 0.69
Control	n (%)	17 (85)	14 (70)	1 (5)	2 (10)	2 (10)	4 (20)	8 (40)
	Titre	2.3 ± 0.32	1.9 ± 0.23	1.6 ± 0.0	2.3 ± 0.29	2.1 ± 0.15	2.2 ± 0.48	2.8 ± 0.46

Lambs were turned out on day 0.

* maternal antibodies.

The lamb weights are presented in Table 4. At the end of the study the weight (mean ± SD) of seropositive and seronegative lambs were 51.4 ± 5.96 and 51.8 ± 5.87, respectively. No significant difference in live weight was found on any sampling day between treated and untreated lambs (P > 0.05).

**Table 4:** Live weight (mean ± SD) of treated and untreated lambs on tick-infested pasture from turnout (day 0)

**Day**	**0**	**28**	**56**	**84**	**112**	**126**
Treated	16.3 ± 2.12	26.4 ± 2.97	34.8 ± 3.03	43.2 ± 3.82	50.6 ± 4.42	52.6 ± 4.61
Controls	16.1 ± 2.54	26.2 ± 3.81	33.9 ± 5.24	41.7 ± 5.53	48.6 ± 6.04	50.8 ± 6.86
Difference (kg)	0.2	0.2	0.9	1.4	2.0	1.8

## Discussion

In the present study, clinical disease was not observed except for fever. Only a few lambs with high fever (≥41°C) typical for TBF were detected [1,4]. This could be due to variants of *A. phagocytophilum* involved [15,16]. However, the animals were only examined every fourteen days and the fever period in *A. phagocytophilum* infected sheep normally lasts around one week [4]. In comparison, most primary infections in the field are not observed [17].

Only a few ticks were found on the animals. The reason for this is unknown, but tick may have attached and detached unnoticeably, since the attachment period on animals for all stages of *I. ricinus* ticks is normally less than 14 days [18]. In the present study, ticks were not observed on 20% of the lambs that seroconverted to *A. phagocytophilum*. Attached ticks may also have been overlooked, since a detailed inspection of the whole animal was not performed. However, most *I. ricinus* ticks on sheep are attached on the head, axillae and inguinal regions [12,13]. In addition, most ticks were found in May/June and in August/September. Earlier studies indicate that cases of TBF in the same county have a similar distribution with 60.4% occurring in May/June and 27.7% in September/October [19].

Maternal antibodies were found in 88% of the lambs. In the previous year, maternal antibodies were found in 89% of the lambs (Stuen, unpublished results), indicating that *A. phagocytophilum* infection is common on the actual pasture. However, the infection seems to occur mainly in the autumn, since the seroprevalence in medio September was only 35%. In the pyrethroid treated group, 30% of lambs were seropositive, which was slightly less than in the control group (40%), although a significantly higher number of ticks were found on the control lambs. The reason for this apparent discrepancy between tick infestation rate and infection prevalence may be due to an unknown number of infested ticks involved (the lambs were only observed every fourteen days) or variation in both *A. phagocytophilum* variants and infection prevalence in the actual tick population. In an earlier study from the same county in Norway, 24 *msp-4* gene variants of *A. phagocytophilum* were found among 16 lambs during the first grazing season [16]. Serological response may differ significantly between variants of the bacterium [15], whereas some variants may not even give a detectable serological titre (Stuen, unpublished result). Unfortunately, variants of *A. phagocytophilum* were not investigated in the present study.

Ticks were found on pyrethroid-treated lambs. This indicates that frequent treatment every second week is not 100% efficient against tick infestation. The present result is in accordance with earlier studies indicating that ticks can be found on animals already 13–14 days after treatment with either cypermethrin or deltamethrin [7,13]. An earlier field study also supports the present results, since most lambs seroconverted already three weeks after pyrethroid treatment [20]. The main reason for this lack of efficiency is unknown. According to the manufacturer, it may take 1–2 days after tick attachment until therapeutic concentration of pyrethroids in the tick is reached (Enemark, personal information). In addition, an earlier study indicated that sheep infested with ticks at the time of pour-on treatment lost their ticks within two days, which may indicate the kill time and the time for the tick to become detached [13]. Furthermore, treatment of young lambs (≤4 weeks) with flumethrin (Bayticol®, Bayer) may be less effective due to lack of skin fat which in turn impairs the distribution of the compound (Enemark, personal communication). However, the present lambs were more than four weeks old when they were treated.

Acute cases of anaplasmosis were detected in both the control and the treatment group. Transmission of *A*. *phagocytophilum* from vector to host generally occurs between 24–48 hours after tick attachment [21-23], but the pathogen have been detected in the salivary glands of questing ticks and may therefore be transmitted immediately when ticks start feeding [24,25]. The results indicate that *Anaplasma* can be transmitted by *I. ricinus* ticks in the time window between attachment and therapeutic concentration of pyrethroids. However, acaricide resistance cannot be ruled out and must be further elucidated. In the present study, resistance seems rather unlikely since pyrethroids have only been used for one grazing season.

No significant weight difference was observed between treated and untreated lambs or between seropositive and seronegative lambs. In contrast, earlier studies indicate a weight reduction of 1.4-3.8 kg in the autumn between seropositive and seronegative lambs [26,27]. An earlier report also indicates that spring treatment of ewes and lambs with cypermectin pour-on produced 6% more lambs at weaning and the treated lambs were 1.5 kg heavier than untreated lambs [28]. The reason for this discrepancy is unknown, but may be due to small sample size, variants of *A. phagocytophilum* involved, infection rate in ticks, other tick-borne pathogens, immune status and conditions of the lambs, management and environmental factors such as for instance variation in temperature and rainfall [4,29].

The present study indicates that the effect of acaricides on the prevalence of *A. phagocytophilum* is limited. However, acaricide treatment may reduce the losses due to secondary infections. For instance, the incidence of lambs with lameness (tick pyaemia) or sudden death (*Bibersteinia/Mannheimia* septicaemia) has been reduced due to pour-on treatment [20]. The reason for this effect is unknown. Ticks may carry several infectious agents or several variants of the same agent [4]. Animals treated with acaricides may have reduced infection pressure due to a reduced number of ticks on each animal [13]. For instance, microorganisms not already present in the salivary glands of newly attached ticks may be more vulnerable to hosts treated with acaricides. In order to examine the beneficial effect of acaricides on tick infested pastures, the distribution of *A. phagocytophilum* variants and other tick-borne pathogens in *I. ricinus* ticks has to be further elucidated.

## Conclusion

The present results indicate that frequent acaricide treatment do not reduce the seroprevalence to *A. phagocytophilum* on tick-infested pasture, although it reduced the number of ticks on treated lambs.

## Competing interests

The authors declare that they have no competing interests.

## Authors’ contributions

SS, JMDE and BN have designed the experimental study. SS performed the field work, carried out the statistical analysis and drafted the manuscript. KA performed the serology. All authors read and approved the final manuscript.
